# The Ideal Method of Brushing the Teeth*This is a liberal extract from a paper published in full with illustrations in the April “Journal of the National Dental Association.” The writer proposes to carry the dentifrice into the contracted proximal area by a slight rotary motion rather than the long, sweeping motion advocated by some. He also surgically enlarges the proximal entrasures to secure access in pyorrhetic conditions.

**Published:** 1919-05

**Authors:** W. J. Charter


### THE IDEAL METHOD OF BRUSHING THE TEETH*

BY W. J. CHARTER, D.D.S.

We will presume the gums are practically in a normal condition, and we are going to keep them in this same condition. The method I am going to describe will, no doubt, lead some to believe that this method of brushing will injure the gums. For the benefit of those who may be of that opinion, I beg to say that it will not, any more than with any other method of using the tooth brush—as it has been tried out on a great many children with no injury, but to the contrary has done a great deal of good in every case.

* This is a liberal extract from a paper published in full with illustrations in the April "Journal of the National Dental Association." The writer proposes to carry the dentifrice into the contracted proximal area by a slight rotary motion rather than the long, sweeping motion advocated by some. He also surgically enlarges the proximal entrasures to secure access in pyorrhetic conditions.

Again some may say the gums will be pushed back out of the interproximal spaces in a normal mouth. For the benefit of those who may believe this to be true, I will state that it is impossible to make the gums recede where there is a normal alveolar process. As has been stated before there are no just grounds for those claims. I can show a picture of a child's mouth, taken before and two years after this method of brushing was installed, and no change has taken place in the gums, except that of a harder and firmer gum tissue. Nature builds up a firmer gum tissue to withstand the friction caused by the tooth brush. The gums in the mouth of a few people appear to be immune from gingivitis and eventually pyorrhea, but a large percentage of the mouths are subject to the ravages of these diseases, and it is up to the dental profession to practice and teach preventive dentistry as much as possible that pyorrhea and decay may be eliminated to the greatest extent possible. Preventive dentistry has not been taught by the dental profession to any great extent so far, as our time has been occupied by the curing of mouth ills, but the time is coming, and not very far off, when we will be devoting a great deal of our time to the prevention of mouth diseases, instead of curing the same.

The virtue of this method of brushing can be better proven in mouths of people where pyorrhea has started, especially in advanced cases where the gum is soft and the alveolar process disintegrated this condition leaves open interproximal spaces which make it easy to place the bristle between the teeth.

The real cause of either dental caries, or pyorrhea has never been found, but it may be assumed that any irritant, of whatever nature which impairs the integrity and continuity of the gingival gum margin may cause pyorrhea.

The irritation which may dissolve the integrity of the gingival border may be presented in various forms, such as calcareous deposits, the vicious use of banded crowns, poorly fitted clasps or retainers on partial dentures, improperly finished fillings, etc.

I wish to state that in almost all cases of medium, or advanced cases of pyorrhea, and this condition exists in almost every mouth of over 30 years, and for the benefit of those who are skeptical I would suggest, that when you return to your offices that you examine with a sharp explorer the first 50 patients that present themselves for any kind of mouth service, and note if the statement I have just made is correct. In making these examinations see whether or not the alveolar process is flat or concave, labio-lingually and bucco-lingually in many cases where it originally was convex. Now, it has been observed that in these cases ideal brushing will render the gingiva hard, in fact so hard and tough that you are unable to crowd it back any farther with any method of brushing and still you have the concave surface of the alveolar process.

At present there is no method of brushing that will reach this surface just referred to, so what is the result? We must resort to surgery and remove the gum and process to be in line or even lower than the center of the interproximal surface labio-lingually or bucco-lingually. This is a very simple operation and I believe it absolutely necessary in keeping the mouth clean. It must be remembered the tooth brush does not remove the film deposit from the mouth that has been brushed from the teeth, but on the contrary with the aid of the saliva and dentifrice, the food film has been changed into a liquid form, and with an abundance of water it is very readily washed out.

In my belief there is no medicament that can be used in the mouth without deleterious effects that will cure pyorrhea, and if this statement is true there is no other course to pursue than to wash out the bacteria which may be easily accomplished, providing we have eliminated the inaccessible pockets, by taking a mouthful of warm water, then by bringing into occlusion the teeth and lips, forcing the water back and forth between the teeth. Use from one to two glasses of water for this purpose. Remember we should wash the bacteria out of the mouth rather than attempt to destroy them medicinally. You can not injure the gum tissue by this method of brushing the teeth. The pyorrhea has already done a great deal of damage. You can not begin to do the damage that the pyorrhea is doing. As we understand it, 90 per cent. of the mouths already have or will have pyorrhea. If that is true, those teeth are going to be lost sooner or later and the disease may also do a great deal of damage systemically to the patient. Why talk about the bristle harming the gum when the pyorrhea is going to cause the loss of the teeth sooner or later? You can not injure the gum tissue with a brush used in this manner.

The diseased gum and process should be removed so as to eliminate all pockets. It is much better to have large spaces between the teeth and roots so that bristles can polish the tooth surfaces that have been affected than to have inaccessible pockets. I remove the process to make it convex between the teeth, instead of concave. There is no brush that will remove the food particles from under such gums, so we must remove the gum which is unsupported by normal alveolar process. Don't allow yourself to believe that after you treat pyorrhea pockets they will fill in with bone tissue to a normal condition. If you do you will be greatly disappointed.

I anesthetize the gum, and with special shape lancets bent at right angles, and with cutting surfaces at different angles, which allows cutting forward or backward, I am able to remove a "v" shape piece of gum buccally or lingually in the interproximal space or between the roots of the molars. In removing the gum I expose the alveolar process. Then with small plain fissure burs I remove the plates of process to a level with the process between the teeth. By doing this there will be no concave surfaces between the roots tq act as receptacles for food. Then pack cotton, saturated with uroform paste between the roots. Repeat every day for two or three days, or until the gum tissue has reformed over the process. The surfaces will be tender to the tooth brush for a short time only.

I don't believe it is possible to teach a class of children in a short time to brush their teeth correctly. You can not teach this method of brushing the teeth by standing up before and talking to children. You can show them how to take the tooth brush in their hands and place it in the mouth, and you can tell them to brush up and down and so on. But if you will notice, there is probably a third of the surface that is not touched. If that is all you are going to teach, it is easy to teach children, but you have neglected one-third of the surfaces of the teeth, and the surfaces that need the brushing most. If we are going to make the teeth clean, we must exert friction on the surfaces that need attention. As I believe you can not get the bristles in between the teeth unless you place them there, and you can not get them there with a sweeping motion. I will admit that this method is very difficult to perform. In fact, I can not teach it in one sitting. It will take ten, or fifteen, or possibly more, lessons, but I keep at it until I do get my patients to do it thoroughly. The tooth brushes are brought to the office and kept in test-tubes and the patients come every day or every other day. If they show reasonable skill I will keep them coming until they accomplish the method. If I can not teach them, then they are dismissed that no valuable time be misspent.

There is one objection to this method and that is it is hard to accomplish. If you can teach people to play the piano, why can we not teach them to clean the teeth correctly? In order to clean the teeth we must come into actual contact with them, and you can not do that with the sweeping motion because there are too many small spaces. Short teeth are difficult to brush, but they are difficult with any method. You must get between the teeth and try and work the bristles into the V-shaped spaces between the teeth. Crowd the bristles down on to the gum. The gums and interproximal spaces require a lot of friction. The more friction the firmer the gum. You can not brush the gum away from the interproximal spaces where it is normal or healthy.

Referring to cases where there are pyorrhea pockets, there is no method of brushing that will clean these pyorrhea pockets to any extent, before they have been trimmed. That surface must be cleared and how are we going to clean it unless we make the surface accessible to the brush. Cut the gum away that we may make friction on the roots of the tooth. Otherwise you can not clean them. If you have a necrosed process standing up between the teeth you have a concave surface between the teeth and that will be hard to clean. Make it convex, so it is easier to get the bristles in.

As I said before, I keep on teaching children and my patient until they grasp the idea, but you can not tell patients how to brush their teeth and have them go on and brush them properly, unless you have actually done the brushing in their mouths. I have tried teaching without demonstrating, getting poor results, but now with this method of teaching and brushing I am getting wonderful results. Try it and you will be convinced.

